# Supplementary material for: Urinary polycyclic aromatic hydrocarbon (PAH) metabolite concentrations in three pregnancy cohorts from 7 U.S. study sites
Source: PLoS One. 2024 Jul 3;19(7):e0305004. doi: 10.1371/journal.pone.0305004 (PMC11221841; doi:10.1371/journal.pone.0305004)
Supplement: S1 Fig — (PDF) [file pone.0305004.s001.pdf]

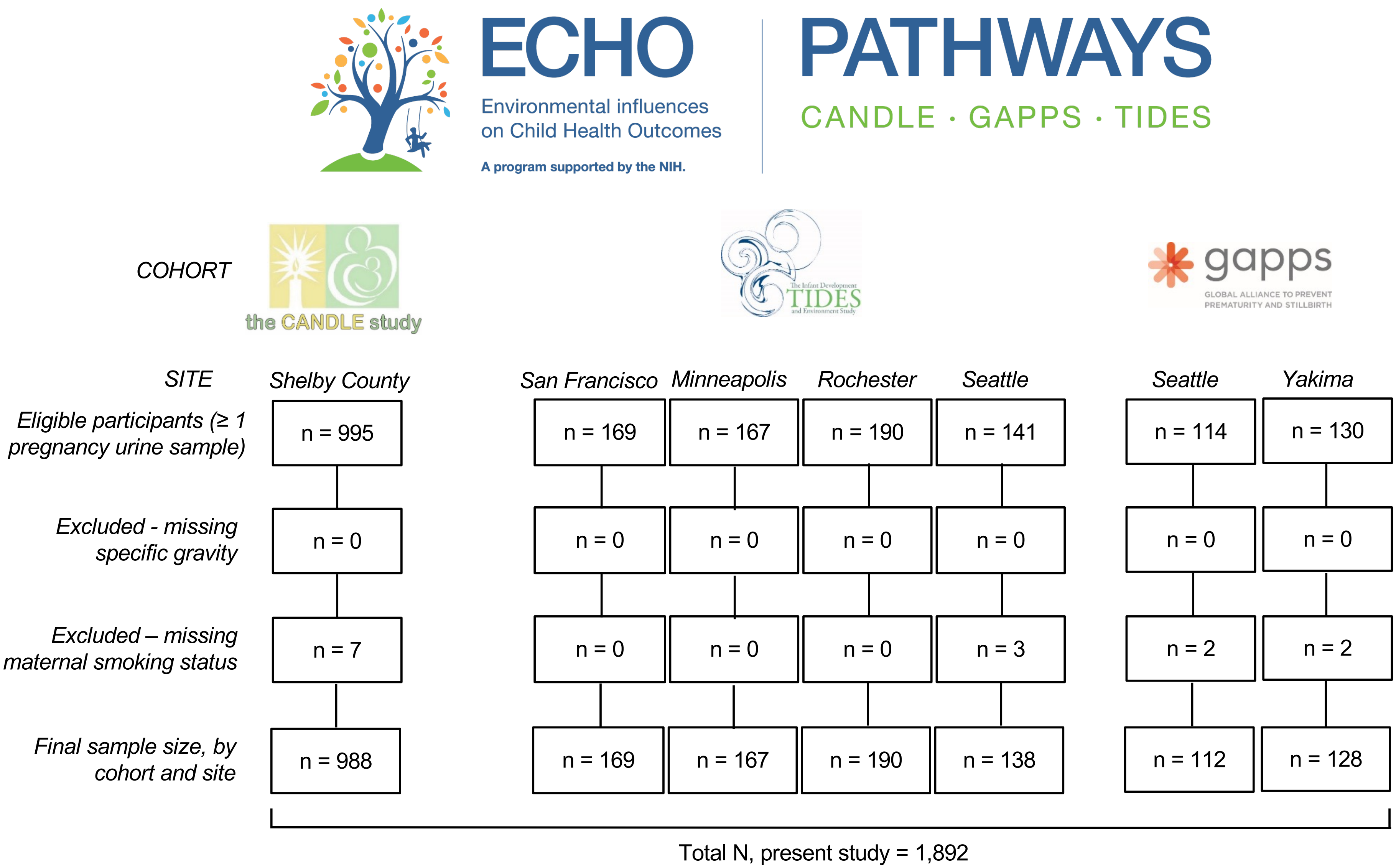

S1 Fig. Participant flow diagram, by cohort and site.
