# Supplementary material for: Urinary polycyclic aromatic hydrocarbon (PAH) metabolite concentrations in three pregnancy cohorts from 7 U.S. study sites
Source: PLoS One. 2024 Jul 3;19(7):e0305004. doi: 10.1371/journal.pone.0305004 (PMC11221841; doi:10.1371/journal.pone.0305004)
Supplement: S2 Fig — Upper panels (salmon) are non-smokers; lower panels (turquoise) are smokers. 1-hydroxypyrene detection frequencies were 89% for non-smokers (n = 885) and 97% for smokers (n = 103). (PDF) [file pone.0305004.s002.pdf]

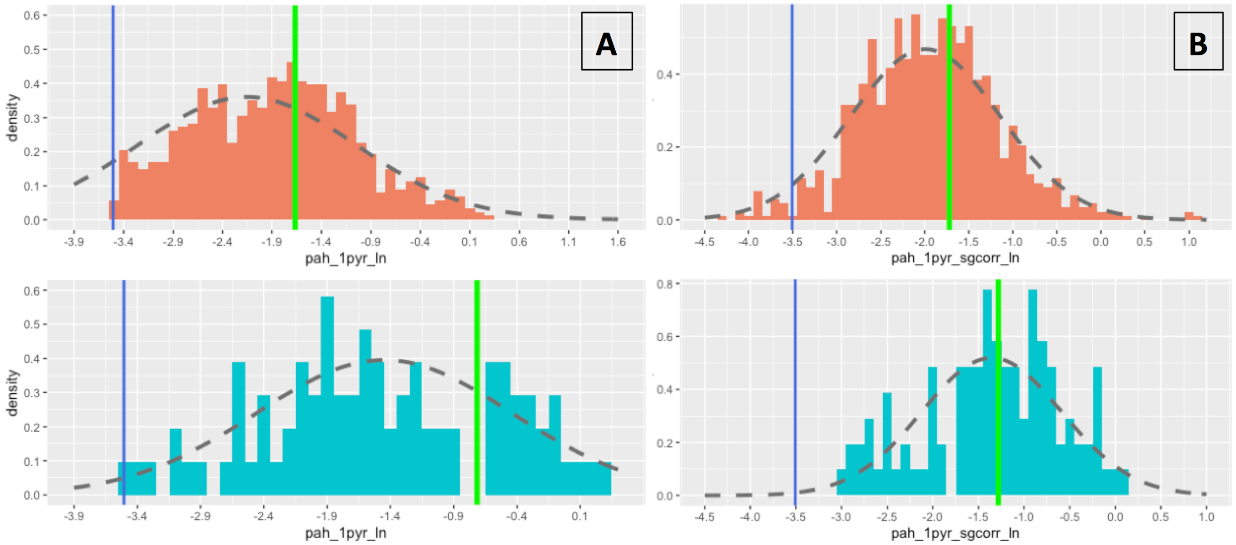

S2 Fig. MLE fitted distributions (dashed line), histograms, estimated modes (green vertical line), and analytical detection limits (blue vertical line) of log-transformed uncorrected (S2A Fig) and specific gravity-corrected (S2B Fig) urinary 1-hydroxypyrene among CANDLE participants. Upper panels (salmon) are non-smokers; lower panels (turquoise) are smokers. 1-hydroxypyrene detection frequencies were 89% for non-smokers (n=885) and 97% for smokers (n=103).

INTERPRETATION: In these figures, the peaks of the MLE fitted distributions appear close to the modes, which we interpret as meaning that the MLE distributions are reasonable fits to the low- to moderately left-censored (i.e., 5-10%) CANDLE data (S2 Fig.) as well as to the more highly left-censored (i.e., 30-40%) GAPPS (Seattle) data (S3 Fig.).
