# Supplementary material for: Urinary polycyclic aromatic hydrocarbon (PAH) metabolite concentrations in three pregnancy cohorts from 7 U.S. study sites
Source: PLoS One. 2024 Jul 3;19(7):e0305004. doi: 10.1371/journal.pone.0305004 (PMC11221841; doi:10.1371/journal.pone.0305004)
Supplement: Attachment — Submitted filename: Masterson et al - Response to reviewers.docx [file pone.0305004.s011.docx]

**Masterson et al. - Response to Reviewers**

*Reviewer #1: I would like to express my gratitude for the opportunity to review your manuscript titled " Urinary polycyclic aromatic hydrocarbon (PAH) metabolite concentrations in three pregnancy cohorts from 7 U.S. study sites". Polycyclic aromatic hydrocarbon (PAH) is a class of chemicals that hold significant environmental importance. It is intriguing to see an updated study on the between urinary PAH and pregnancy cohort. The study is well written. Consequently, I have some questions and suggestions that need to be addressed:

Abstract: The abstract could be organized using subheadings such as "Objective," "Methods," "Results," and "Conclusion."*

Thank you for the recommendation-- we added the suggested subheadings to the abstract.  *Methods: Since this study was used to data set, I assume it involved multiple data sets. It is highly recommended that you clarify how many cycles' data sets were merged and provide information on the sample size of each data set. Additionally, please add the flow chart accordingly to reflect these details.*

Thank you for the suggestion. We made a flow chart and added it to the manuscript as Figure S1 in the Supporting Information. We also added this sentence to the Participants section of Materials and Methods:

"Figure S1 in the Supporting Information shows the participant flow diagram by cohort and site."

*"Urinary Polyaromatic Hydrocarbon: Please provide information on how the samples were stored and at what temperature."*

Thank you for catching this. We added the following sentence to the beginning of the OH-PAH Methods section:

"Samples were stored at -80°C in each cohort's respective biorepository until analysis for OH-PAHs at the New York State Department of Health, Wadsworth Laboratory."

*Discussion section: The discussion should emphasize the novelty of your research results by comparing them with findings from previous studies. It would be beneficial to delve deeper into the interpretation of the results and provide a more comprehensive analysis.*

Thank you for this constructive feedback-- please see related comment and our response below.

*There are few studies on same population and it is recommended to cite the following studies in this study:* [*https://www.sciencedirect.com/science/article/abs/pii/S0013935122001025*](https://urldefense.com/v3/__https:/www.sciencedirect.com/science/article/abs/pii/S0013935122001025__;!!K-Hz7m0Vt54!mTTWvB05Eqvkfmlp6AvcgALG-yEhySiXreoPTwTigVxfA4qcnTqoZ-gZB8C3yFreQsrNY3fuS_ygo9w$)*,* [*https://academic.oup.com/inthealth/article/15/2/161/6617857*](https://urldefense.com/v3/__https:/academic.oup.com/inthealth/article/15/2/161/6617857__;!!K-Hz7m0Vt54!mTTWvB05Eqvkfmlp6AvcgALG-yEhySiXreoPTwTigVxfA4qcnTqoZ-gZB8C3yFreQsrNY3fuS_L8tE4$)*,* [*https://link.springer.com/article/10.1007/s10653-022-01438-y*](https://urldefense.com/v3/__https:/link.springer.com/article/10.1007/s10653-022-01438-y__;!!K-Hz7m0Vt54!mTTWvB05Eqvkfmlp6AvcgALG-yEhySiXreoPTwTigVxfA4qcnTqoZ-gZB8C3yFreQsrNY3fuPGlAJXs$)*.*

Thank you for the suggestions. We added the recommended citations to a new paragraph we added to Statistical Methods (see related comment below): "Since income and education were previously associated with urinary OH-PAH concentrations in pregnant (Cathey et al. 2018) and non-pregnant people (Mallah et al. 2023a, 2023b; Mallah et al. 2022; Liu and Jia 2016), we compared GM/GSD OH-PAH concentrations across income and education categories..."

*Overall, the manuscript would benefit from improvements in the English language. I recommend having it edited the language quality with professional standards.*

Thank you for the feedback. Senior scientists from our research group who are native English speakers with extensive publication records, have carefully reviewed this revised version of our manuscript for language use.

*Reviewer #2...Masterson et al. measured and analyzed 12 PAH metabolites in the urine of pregnant women living in the United States from seven metropolitan sites in the ECHO PATHWAYS Consortium.

The data collected in this study can be useful for evaluating the relationship between prenatal exposure to PAH exposure and health outcomes and evaluating sources of exposure (e.g., planned studies). However, without a more detailed analysis of the exposure data, it is unclear how this study per se advances the science, as urinary data on women of reproductive age is available from NHANEs. [this study doesn’t can’t evaluate differences due to pregnancy as samples collected before pregnancy are not available}.

From my perspective, the study would be more informative and public health-relevant if it conducted a deeper dive to explore differences in urinary PAH levels.*

Thank you for this constructive feedback. The purpose of this study is to describe and characterize OH-PAH concentrations in pregnancy among participants in the large ECHO/PATHWAYS consortium. This data is a unique characterization of urinary PAH exposure during pregnancy given few women in the NHANES database were pregnant during sampling times. We are currently preparing a separate manuscript reporting results of analyses *emphasizing sources of exposure* where we formally test the hypothesis that exposure to environmental tobacco smoke, measured via urinary cotinine, is associated with urinary OH-PAHs in the pooled data. The new models are adjusted for specific gravity, site, season, year, analytical batch, and body mass index, and for SES characteristics such as household income, education, employment status, and neighborhood deprivation index. In similarly-adjusted models, we are also formally testing the hypothesis that ambient air pollution, estimated via 24-hr average PM_2.5_ concentrations at participants' residential census tracts (available from CDC's Environmental Public Health Tracking Network), is associated with urinary OH-PAHs.

We prefer to keep the present manuscript descriptive in nature, so that we can present a detailed picture of OH-PAH levels in this large study sample. We believe this will be useful to others examining OH-PAH concentrations among pregnant people in the United States.

To satisfy the reviewer's curiosity about race/ethnicity patterns in the present analyses, however, we conducted *post hoc* exploratory race/ethnicity analyses -- see related response below.

*Some suggestions are as follows.*

*• SES/Education: conduct a formal analysis (rather than just providing data stratified by SES) to determine the relationship between SES and urinary PAH metabolite levels*

Thank you for the suggestion. There is indeed evidence in the literature that both income and education are associated with urinary OH-PAH levels in both pregnant and non-pregnant people. This was the reason we chose to highlight these characteristics in Table 3. In response to the suggestion, we conducted additional analyses of urinary OH-PAH concentrations by income, and separately, education category, by cohort and site, using Tobit regression to account for left censoring in the data and adjusting for urinary specific gravity.

We added the following new paragraph describing these analyses to Statistical Methods:

"Since income and education were previously associated with urinary OH-PAH concentrations in pregnant (Cathey et al. 2018) and non-pregnant people (Mallah et al. 2023a, 2023b; Mallah et al. 2022; Liu and Jia 2016), we compared GM/GSD OH-PAH concentrations across income and education categories. We also used Tobit regression to formally evaluate associations between income and education and log-transformed OH-PAH concentrations, accounting for left censoring in the data and adjusting for urinary specific gravity. In these regressions, income was modeled following the recommended approach for multi-site PATHWAYS analyses: harmonized household size (2-3, 4, 5, ≥ 6 people) and income (adjusted for region and inflation to 2012 $USD) from the 4-6 year old visit instead of the prenatal visit because of the proportion of right-censored prenatal TIDES and GAPPS observations, which was addressed at the 4-6 year old visit with additional upper income categories. The models included natural log household income interacted with household size ('household size adjusted household income'), and the corresponding main effects. For the education regressions, the "< High school" and "High school completion" categories were combined to avoid low cell counts in the "< High school" category. Complete case analysis was used for the Tobit regressions."

We also changed "...(including annual household income, ..." to "...(including annual household income at enrollment, ..." the Cohort characteristics section of Methods to make it clear that the descriptive statistics shown in Table 1 apply to household income at enrollment (and not the 4-6 year old visit).

We also changed the text of the Socioeconomic characteristics section of Results from:

"Table 3 shows s.g.-corrected GM OH-PAH concentrations stratified by site and socioeconomic characteristics (income and education). We did not formally test the following observations. We observed some trends between lower concentrations of most OH-PAHs and higher income and higher education levels. These varied by site and metabolite. Most consistently, we observed lower concentrations of 2-hydroxynaphthalene among those with higher education levels at all sites. By contrast, OH-PAH concentrations did seem to vary by income at some sites but consistent patterns were not apparent..."

to:

"Table 3 shows s.g.-corrected GM OH-PAH concentrations stratified by site and socioeconomic characteristics (income at enrollment and education). Tables S3 and S4 in the Supporting Information show results from the Tobit regressions of log OH-PAH concentrations on income and education, respectively, adjusted for s.g.. In these analyses, income was not consistently associated with OH-PAH concentration for any analyte or site except for GAPPS Seattle, where concentrations increased with household size adjusted household income, although the small sample size of the complete case analysis (n = 51) limits interpretation (Table S3). Among CANDLE and TIDES Rochester participants, OH-PAHs were lower in the higher two education categories compared to "< High school/high school completion" but this trend was not consistently observed among participants at the other sites."

Last, we changed the Socioeconomic characteristics section of Discussion from

" Among the PATHWAYS participants who did not smoke during pregnancy, we observed patterns in income and/or education level related to OH-PAH concentrations. These patterns varied by study site and metabolite but we did not formally test these differences as our goal was to be descriptive, rather than to test group differences."

to:

"Among the PATHWAYS participants who did not smoke during pregnancy, we observed inconsistent patterns in income and/or education level related to OH-PAH concentrations."

*• Age: conduct a formal analysis to determine the relationship been age and urinary PAH metabolite levels.*

Thank you for the suggestion. We did not find prior published evidence of associations between age and OH-PAH concentrations in pregnant people so we did not formally test age thus prefer to leave it out of the manuscript. In response to this suggestion, however, we conducted *post hoc* analyses of urinary OH-PAH concentrations by continuous age (years), by cohort and site, using Tobit regression to account for left censoring in the data and adjusting for urinary specific gravity. *P*-values from these analyses are summarized below:

*• Results should note differences in cohorts by age (e.g., women in SF and Seattle, Memphis, and Rochester are younger).*

Thank you for the suggestion. In response, we changed this sentence in Results, "... The mean age was 29 (SD: 6) years, with variation across sites..." to " The mean age was 29 (SD: 6) years, with variation across sites; in general, CANDLE, TIDES Rochester, and GAPPS Yakima participants were younger than participants at other sites."

*• Race/Ethnicity: Provide the racial/ethnicity group in Table 1, stratify PAH levels by race/ethnicity, and conduct a formal analysis.*

In the present study, as well as the new analyses described above, we did not formally test the social constructs of race/ethnicity because we have no *a priori* reason to believe that urinary OH-PAHs differ by race/ethnicity in a manner independent from SES, residence location, environmental tobacco smoke exposure, and other factors included in our new analyses in preparation. In the present study, both race and ethnicity were associated with income and education in non-parametric Kruskal-Wallis tests, however not consistently across sites. Theoretically, there could be race/ethnicity differences in dietary PAH intakes, physiological processes influencing PAH toxicokinetics, or other factors, however empirical evidence for these is scant so we do not include race/ethnicity in the new models.

In response to the reviewer's suggestion, we conducted *post hoc* analyses of urinary OH-PAH concentrations by race (Black/African American [referent category], White, and Other) by cohort and site, using Tobit regression to account for left censoring in the data and adjusting for urinary specific gravity. *P*-values from these analyses are summarized below:

We also conducted *post hoc* analyses of urinary OH-PAH concentrations by ethnicity (Not Hispanic/Latino [referent category], Hispanic/Latino) by cohort and site, using Tobit regression to account for left censoring in the data and adjusting for urinary specific gravity. *P*-values from these analyses are summarized below; note that these analyses are not corrected for multiple comparisons:

*• Geographical location: Are any participants from rural or suburban areas outside the city? If so, this could be explored.*

Thank you for this helpful suggestion and we agree that urban vs. rural (or suburban) differences in urinary OH-PAH concentrations is an interesting scientific question because of the potential influence of urban air pollution. In our upcoming manuscript (in preparation) on PAH exposure factors in the ECHO/PATHWAYS consortium, we used census-tract level estimates of ambient particulate air pollution (PM_2.5_) from CDC's Environmental Public Health Tracking Network to represent PAH exposures via ambient air pollution. To some degree, this analysis captures urban vs. rural differences since some of the census tracts included are more urban and others more rural. On the other hand, other important PAH exposure sources such as environmental tobacco smoke and diet may or may not vary with urban vs. rural residence location. We examine these potential exposure factors as well in the upcoming manuscript.

*• Consider summing the metabolites for the analyses by age, SES, race/ethnicity, and possibly geographical location.*

Thank you for the suggestion. We know that some researchers sum PAH metabolites but we do not do this in part because the parent PAHs can come from different sources. For example, 1-hydroxynaphthalene is a metabolite of both naphthalene and the ubiquitous insecticide carbaryl, while 2-hydroxynaphthalene is only a metabolite of naphthalene. Because of this, summing the naphthalene metabolites could provide a misleading estimate of naphthalene exposure, since some portion of the sum may be attributable to carbaryl instead of naphthalene.

Similarly, we would not sum naphthalene and pyrene metabolites, for example, since the parent compounds are quite different in terms of their physicochemical properties. Naphthalene behaves more like a volatile organic compound and pyrene behaves more like a semi-volatile organic compound in terms of environmental transport and fate. Since physicochemical properties also influence toxicokinetics (e.g., half-life in the human body), a single spot urine sample could theoretically provide a snapshot of naphthalene and pyrene exposures from two different time points, if one compound is metabolized more quickly than the other.

Last, the parent PAHs we considered may also have different modes of toxicity. For example, the U.S. Environmental Protection Agency's Integrated Risk Information System (EPA IRIS) lists different toxicological end points supporting the oral reference doses for naphthalene (decreased body weight in males), fluorene (decreased red blood cells, packed cell volume and hemoglobin), and pyrene (kidney effects) (note: there is currently no oral reference dose for phenanthrene). For these reasons, we prefer to characterize the OH-PAHs separately.

EPA IRIS References:

U.S. EPA Integrated Risk Information System. Fluorene. https://iris.epa.gov/AdvancedSearch/?keyword=fluorene; accessed 4 Oct 2023.

U.S. EPA Integrated Risk Information System. Naphthalene. https://iris.epa.gov/ChemicalLanding/&substance_nmbr=436; accessed 4 Oct 2023.

U.S. EPA Integrated Risk Information System. Pyrene. https://iris.epa.gov/ChemicalLanding/&substance_nmbr=445; accessed 4 Oct 2023.

*Minor comments
• Figure 2: format the graph for Candles to be consistent with the other sites.*

Thank you for noticing this difference. CANDLE only contains one site (Shelby County, Tennesee) so there are no comparison data for the y-axis correlations. The other panels show the correlations for two different sites within the same cohort, namely TIDES (Seattle) and TIDES (Rochester), TIDES (Minneapolis) and TIDES (San Francisco), and GAPPS (Yakima) and GAPPS (Seattle). We were trying to be efficient with space and see how readers might be confused by the presentation. We decided to keep the figure as is, rather than include 3 additional panels of single site correlations (like the CANDLE panel), to save page space.

*• Repeated measures across pregnancy. How would changes in the Glomerular filtration rate during pregnancy affect urinary concentrations of PAHs metabolites?*

This is a good question as there are many physiological changes across pregnancy that potentially affect toxicant absorption, distribution, metabolism, and excretion. Increasing GFR could theoretically mean faster excretion of naphthalene, fluorene, phenanthrene and pyrene in, say, the 2nd trimester compared to the 1st trimester, for a given exposure. For example, a 1st trimester spot urine sample collected at 2PM may capture PAHs inhaled during the morning commute, while a 2nd trimester 2PM sample may have missed morning commute exposures as the corresponding OH-PAHs were excreted hours earlier.

Ideally, urinary biomarker concentrations would be multiplied by urinary flow rates to get more directly comparable measurements of mass excreted over time (MacPherson et al. 2018). In practice, urinary flow rates are rarely measured because of the burden of asking participants to collect and store urine over periods such as 24 hours, thus researchers typically rely on spot urine samples and account for hydration status using specific gravity or creatinine adjustment.

To acknowledge this limitation, we added the following phrase to the Repeated Measures section of the Discussion:

"The low ICCs we observed across the repeated measures within each TIDES site may illustrate the limitations of using a single spot urine measurement to represent typical PAH exposures but may also be related to the potential influence of season relative to pregnancy stage, to additional variability introduced by changes in glomerular filtration rate and/or other physiological processes across pregnancy (MacPherson et al. 2018), or to some combination of factors."

New reference added:

MacPherson S, Arbuckle TE, Fisher M. Adjusting urinary chemical biomarkers for hydration status during pregnancy. J Expo Sci Environ Epidemiol. 2018;28(5):481-493. doi: 10.1038/s41370-018-0043-z. PMID: 29880833; PMCID: PMC8075920.
