# Supplementary material for: Urinary polycyclic aromatic hydrocarbon (PAH) metabolite concentrations in three pregnancy cohorts from 7 U.S. study sites
Source: PLoS One. 2024 Jul 3;19(7):e0305004. doi: 10.1371/journal.pone.0305004 (PMC11221841; doi:10.1371/journal.pone.0305004)
Supplement: Attachment — Submitted filename: Masterson et al - Response to reviewers - March 2024.docx [file pone.0305004.s012.docx]

**Masterson et al. - Response to Reviewers**

PONE-D-23-14326R1

Urinary polycyclic aromatic hydrocarbon (PAH) metabolite concentrations
in three pregnancy cohorts from 7 U.S. study sites

PLOS ONE

Review Comments to the Author

*Reviewer #1: Manuscript is well written and can be accepted for publication. Comments are well addressed and revised manuscript accordingly.*

Thank you—we appreciate the constructive comments & suggestions from Reviewer #1 throughout the review process.

*Reviewer #2: While the authors have made some improvements in the revised manuscript, my original comment –the study would be more informative and public health-relevant if the authors conducted a deeper dive to explore differences in urinary PAH levels–remains. The authors state that they prefer to keep this manuscript and are preparing a separate analysis of the exposure data. This new manuscript sounds exciting. The authors should provide a rationale for what is novel about the current study and why it warrants publication…*

Thank you and we agree the new manuscript (Riederer et al., *in preparation*) is exciting (because it shows consistent associations between environmental tobacco smoke exposure and urinary OH-PAHs in the non-smoking pregnant ECHO/PATHWAYS participants).

We believe that the present descriptive paper (Masterson et al.) is novel because of its large sample size—the largest study of urinary OH-PAHs in pregnant people to date that we are aware of—its geographic diversity (multiple sites across the USA), and our use of maximum likelihood estimation distribution fitting to impute non-detects instead of a single value (e.g., LOD/sqrt(2)), which can bias estimates of central tendency. We thank Reviewer #2 for pushing us to include the exploratory race and ethnicity analyses in the main manuscript which we have now done and hope makes the paper more interesting to readers. Please see our related responses below, and thank you again to Reviewer #2 for such a thoughtful critique.

*I appreciate that the authors conducted in response to my comments (e.g., SES, race/ethnicity); however, they only included the SES analyses in the revised manuscript. The analyses by race and age may help improve the utility of the manuscript as there appears to be differences in PAH metabolite concentrations by race in the largest cohort (which the authors state has the higher number of Black women). The authors suggest they may explore this in more detail in their new paper, but then again, what is the novelty of this paper?*

*I recommend the authors report the racial/distribution in Table 1 and include the age and race/ethnicity/age analysis in this paper. I did not understand why Black/African-American was the referent group…*

Thank you for the recommendation; we added the race and ethnicity distributions to Table 1 (with “White” as the referent group, since it was the largest group).

We also moved the exploratory race and ethnicity analyses into the paper, specifically:

- We added “race and ethnicity” to the “Cohort characteristics” section of Methods and added a new sentence to the “Statistical analyses” section of Methods: “We also explored associations with…race (White, Black/African American, and an “Other” category comprised of Asian, Native Hawaiian/Pacific Islander, American Indian/Alaska Native, Other, and Multiple to avoid low cell counts), and ethnicity (Not Hispanic/Latino, Hispanic/Latino) using the same approach.”
- We added the geometric means and geometric standard deviations by analyte, site, and race and ethnicity categories to Table 3.
- We added a new Table S6 (“Maternal age coefficients from Tobit regressions of log urinary OH-PAH concentrations vs. urinary specific gravity and race, by cohort and site, non-smokers, complete cases”) and Table S7 (“P-values of the maternal ethnicity coefficient in Tobit regressions of urinary OH-PAH concentrations vs. urinary specific gravity and ethnicity, by cohort and site, non-smokers”)
- We added a new sentence summarizing the exploratory findings with race and ethnicity to the “Socioeconomic characteristics” section of Results: “Race was not associated with OH-PAHs at most sites except CANDLE and TIDES Rochester. In CANDLE, 1- and 2-hydroxynaphthalene, 2/3/9-hydroxyfluorene, 2- and 3-hydroxyphenanthrene was higher in Black/African American vs. White participants, and 1-hydroxypyrene was higher in both Black/African American and Other participants compared to White participants. In TIDES Rochester, 2-hydroxynaphthalene was higher in both Black/African American and Other participants compared to White participants, and 2- and 3-hydroxyphenanthrene and 1-hydroxypyrene were higher in Black/African American vs. White participants (Table 3; Table S6). Ethnicity was not consistently associated with OH-PAH levels at any site (Table 3; Table S7). ”
- We added race to this sentence in the abstract, “Among non-smoking participants, we observed some patterns by income, education, and race but these were not consistent and varied by site and metabolite.
- We added race to this sentence in the “Socioeconomic characteristics” section of Discussion: “…we observed inconsistent patterns in income, education, and race related to OH-PAH concentrations…”

*The rationale for not including age in the paper– We did not find prior published evidence of associations between age and OH-PAH concentrations in pregnant people, so we did not formally test age and thus prefer to leave it out of the manuscript– is not adequate to me. The purpose of research is to explore new hypotheses and ideas.*

Thank you for the recommendation—in response, we moved the age analysis into the paper, specifically:

- we added a new sentence to Methods: “We also explored associations with maternal age in years… using the same approach…”
- we added a new Table S5 (“Maternal age coefficients from Tobit regressions of log urinary OH-PAH concentrations vs. urinary specific gravity and continuous age (years), by cohort and site, non-smokers, complete cases”)
- we added a new sentence summarizing the exploratory findings with age to the “Socioeconomic characteristics” section of Results: “Age was not consistently associated with OH-PAH levels for any site except for CANDLE where each year of age was associated with statistically significantly lower levels of 1- and 2-hydroxynaphthlane, 2/3/9-hydroxyfluorene, 3-hydroxyphenanthrene, and 1-hydroxypyrene (Table S5).”
